# Retrospective Attention Interacts with Stimulus Strength to Shape Working Memory Performance

**DOI:** 10.1371/journal.pone.0164174

**Published:** 2016-10-05

**Authors:** Theresa Wildegger, Glyn Humphreys, Anna C. Nobre

**Affiliations:** 1 Department of Experimental Psychology, University of Oxford, Oxford, United Kingdom; 2 Oxford Centre for Human Brain Activity, University of Oxford, Oxford, United Kingdom; University of Groningen, NETHERLANDS

## Abstract

Orienting attention retrospectively to selective contents in working memory (WM) influences performance. A separate line of research has shown that stimulus strength shapes perceptual representations. There is little research on how stimulus strength during encoding shapes WM performance, and how effects of retrospective orienting might vary with changes in stimulus strength. We explore these questions in three experiments using a continuous-recall WM task. In Experiment 1 we show that benefits of cueing spatial attention retrospectively during WM maintenance (retrocueing) varies according to stimulus contrast during encoding. Retrocueing effects emerge for supraliminal but not sub-threshold stimuli. However, once stimuli are supraliminal, performance is no longer influenced by stimulus contrast. In Experiments 2 and 3 we used a mixture-model approach to examine how different sources of error in WM are affected by contrast and retrocueing. For high-contrast stimuli (Experiment 2), retrocues increased the precision of successfully remembered items. For low-contrast stimuli (Experiment 3), retrocues decreased the probability of mistaking a target with distracters. These results suggest that the processes by which retrospective attentional orienting shape WM performance are dependent on the quality of WM representations, which in turn depends on stimulus strength during encoding.

## Introduction

Working memory (WM)–our ability to maintain information over short periods of time to guide adaptive behaviour—is a fundamental cognitive function that is constrained by its limited capacity, shaping both the quality and quantity of WM contents. Given these capacity restrictions, researchers have been concerned with identifying and characterising variables that determine WM variability within individuals over time.

There are systematic effects of orienting attention retrospectively to individual locations or items within WM: a process termed ‘retrocueing’ [1–3]. The exact mechanisms underlying the retrocueing benefit are still being debated. Studies have examined the mechanisms supporting the retrocueing benefit by fitting computational models to WM performance, and assessing which sources of WM error are reduced following valid retrocues [4–7]. Do valid retrocues improve the representational quality of WM items, increase the likelihood of successfully retrieving the target, reduce the erroneous responses based on non-target items (often referred to as misbinding), or is it a combination of factors? The results from studies so far are somewhat mixed. Some studies report that retrocueing primarily increased the probability of accurate memory recall without influencing the quality of item representation [4], [6], [8]. Others report that retrocueing affects both the probability of correctly remembering a target item and the representational quality of successfully remembered target items [5], [9–12].

A recent report by van den Berg and colleagues [13], comparing 32 WM models, showed that including an additional parameter to account for misbinding of items consistently improved model fits across experiments. While only some retrocueing studies have included a third parameter for misbinding, those that did all reported effects of retrocueing on misbinding [5], [7–9], [11].

To date it remains unclear how and when retrocues affect the probability of accurate target recall, the representational quality of successfully remembered target items, or misbinding. Here, we explore the possibility that the variability in reported retrocueing benefits is related to inherent variability in WM representations. Indeed, the main conclusion from [13] factorial model comparison was that a key aspect of WM performance—how well each item is remembered—is variable across items and trials even for a given set size. Assuming that retrocues act on internal representations in WM, the retrocueing benefit may interact with the representational quality of WM items.

There already exists some support for this proposal. [5] showed that observers flexibly shifted retrocueing strategies along with task demands, with the retrocueing benefit stemming from different mechanisms depending on the reliability of cues. Similarly, [9] found that effects of retrocues on misbinding rates were more pronounced in the presence of interference (i.e. in dual-task conditions). These findings suggest that some of the discrepancies in the retrocueing literature are due to the flexibility of mechanisms supporting the retrocueing benefit: depending on the task details, certain performance patterns emerge because retrocues are used differently to improve various aspects of WM performance. Perhaps, some of the ambiguity regarding the functional mechanism of the retrocueing benefit is related to a flexibility of mechanisms underlying the retrocueing benefit.

While there is clear evidence for variability in the quality of WM representations [13–15], the sources of this variability are not fully understood. It has been proposed that intrinsic noise fluctuations impact on the stability of WM representations [14–17]. In line with this possibility, ongoing fluctuations in neural processing influence not only the detection of an external stimulus [18–20], but also WM recall performance [21]. Complementing the effects of internal, neural noise, fluctuations in bottom-up stimulus properties and viewing conditions (e.g. stimulus contrast or noise levels) during encoding appear to be an obvious possible source of influence on the quality of WM representations. To the best of our knowledge, however, the influence of stimulus strength during encoding on mental representations has not been carefully considered in WM research and is typically left out in contemporary models of WM [14], [15], [22–24].

A couple of findings lend promise to the notion that bottom-up stimulus properties during encoding impact on the representational quality of items in WM. [25] reported that orientation judgments are more accurate for cardinal than for oblique orientations. [26] showed that these effects persist into WM, and also demonstrated that recall performance for colours depended on the specific colour value. At least some of the variability in WM, therefore, appears to be stimulus driven.

The great majority of retrocueing studies to date have used high-contrast stimuli. Recently, however, a study reported effects of ‘retrocues’ on a low-contrast stimulus presented at threshold [27]. Participants were asked to remember an orientation stimulus presented at threshold, and valid or invalid cues were presented before or after stimulus presentation (from -100 ms to +400 ms). After a delay period, responses were made on a continuous scale. Both pre- and retrocues reduced guess rate but did not affect the quality of WM representations, but the effect was least pronounced for cues presented 400 ms post stimulus. Importantly, most retrocueing studies have used much longer delay periods, and it is not clear whether the observed effect would persist beyond 400 ms. Because of the short interval preceding the retrocue, it is ambiguous whether cueing in Thibault’s study occurred within maintenance of iconic or working memory or some combination of both [28], [29]. Furthermore, as only one item was presented, it was not possible to assess effects of retrocueing on misbinding rates. Finally, this study only used one contrast level, with stimuli presented at threshold, so it is not possible to assess systematically the impact of varying contrast levels on the retrocueing benefit.

**Fig 1:**
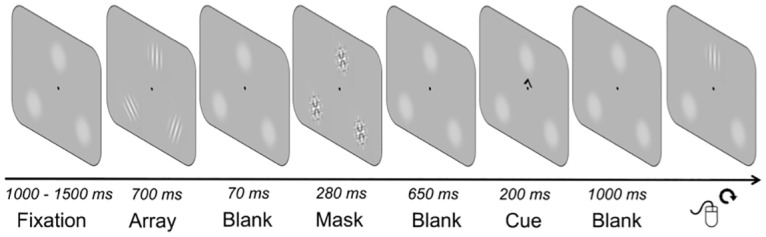
Task design for Experiments 1–3. A central fixation point was presented for a variable duration between 1,000–1,500 ms (randomly determined) followed by a 700-ms stimulus array and, after a 70-ms blank display, a 280-ms backwards-mask display. After another 650-ms blank display, a 200-ms retrocue was presented indicating which of the three stimuli they had to report at the end of the trial. After a final 1000-ms blank display, participants reported the target orientation using the computer mouse.

In our current study, we ask how retrocueing combines with the effects of stimulus strength to modulate WM. We employed the same task (Fig 1) across three experiments in which we varied contrast levels of the to-be-remembered information and manipulated attentional orienting within WM using a retrocueing paradigm [2], [4]. We fit a popular model of WM to our data [30] to estimate the recall precision of target-related responses, the proportion of guesses, and the proportion of misbinding errors. We reasoned that, if effects of retrospective attentional orienting depend on the representational quality of WM items, there should be systematic effects of bottom-up stimulus properties on the retrocueing benefit.

## General Methods

### Participants

The experimental protocols were reviewed and approved by the Central University Research Ethics Committee of the University of Oxford. All participants had normal or corrected-to-normal vision and were naïve to the purpose of the experiment. Participants gave informed written and verbal consent before taking part, and received course credits or financial compensation (£10 per hour) for taking part. The number of participants and inclusion criteria are outlined in the corresponding sections for each experiment.

### Apparatus

Stimuli were created in MATLAB v.7.10 (MathWorks) and presented using the Psychophysics Toolbox [31]. They appeared against a uniform mid-grey background on a 23-inch light-emitting-diode (LED) display. The display had a spatial resolution of 1920x1080 and a refresh rate of 120 Hz. In Experiment 3, a chin rest was used to maintain a constant viewing distance and head position, and eye gaze was recorded binocularly with a desktop-mount video-based eye tracker at 500 Hz (EyeLink 1000, SR Research, Ontario, Canada), using the EyeLink Toolbox extensions for MATLAB [32].

### Task and Stimuli

Participants viewed a briefly presented array of three peripheral, randomly oriented Gabor patches. Their task was to remember the three stimulus orientations. During the delay interval a valid or neutral spatial retrocue was presented. At the end of the trial, participants were probed to report back one of the three orientations. Fig 1 provides a task schematic.

Trials began with a central fixation dot presented for 1,000–1,500 ms (randomly determined from a uniform distribution). The three target stimuli then appeared for 700 ms. After a short blank interval (70 ms), they were followed by masks (280 ms). An interval of 650 ms followed, after which a central retrocue stimulus was presented for 200 ms. After a 1,000-ms blank interval, a probe appeared at one of the three locations. In trials with spatial retrocues (valid condition), the probe appeared at the retrocued location. On trials with neutral retrocues (neutral condition), the probe appeared at one of the three locations randomly determined on each trial. The probe was a Gabor patch (starting at a random orientation), and participants reported the target orientation by rotating the probe stimulus to the remembered target orientation by moving the computer mouse upward (counterclockwise rotation) or downward (clockwise rotation) and then clicking the mouse to confirm a response. Accuracy was emphasized, and participants were encouraged to take as much time as they needed to complete their response. Feedback was provided for 200 ms after the response. Feedback was presented in the form of two small, white dots indicating either end of the target orientation. The subsequent inter-trial interval (ITI) was 500 ms. Note that whereas participants were instructed to perform the task without moving eyes, gaze position was only tracked in Experiment 3.

All stimuli appeared atop 10% contrast luminance pedestals, which were present throughout the entire experiment. The three stimuli in the memory array appeared in a triangular configuration. One stimulus was centred at: 2.6° above the horizontal meridian on the vertical meridian, and the other two were located at 3.1° to the left or right from the vertical meridian and 2.6° below the horizontal meridian (measured from the middle of the stimulus). Target stimuli consisted of Gabor patches (Gaussian-vignetted sinusoidal gratings) with a diameter of 3.5° of visual angle and a spatial frequency of 2 cycles per degree of visual angle. The Gaussian envelope had a space constant of 0.44°. The range of contrast levels for the target Gabor patches varied in each of the three experiments. The stimuli used for backward masking were constructed by applying a Gaussian-vignette to the convolution of 90% contrast square-wave gratings at four orientations of 90°, 180°, 45°, and 135°. The spatial retrocue stimulus (1.59° x 1.59° of visual angle) consisted of a black arrow pointing to one of the three locations which indicated (with 100% validity) which of the three target stimuli was to be reported (valid condition). In the neutral condition, the retrocue stimulus consisted of the outline of a black rectangle presented around fixation. The probe orientation was randomly chosen on each trial.

In all cases, the specific contrast levels used in individual trials were equiprobable and randomly determined, and all contrasts were always the same within a given display. Retrocueing conditions (valid, neutral) were equiprobable and randomized. The orientations of the target stimuli were randomly chosen on each trial.

### Procedure

Participants completed the experiment seated in a dimly lit room. Before starting, they were given a series of training trials (minimum of 48), which were identical to the experimental task, until they were confident they could do the task. During the practice session only the highest contrast level was used. Rest breaks were provided after every block.

## Experiment 1

### Inclusion Criteria

To reduce group variability, we only included participants who performed within 2 SD of the group mean in all conditions (not averaged over contrast). Furthermore, the lowest contrast level in Experiment 1 was included to examine whether attentional selection in WM could raise a stimulus above threshold that otherwise would have been below-threshold for WM reports [27], [33]. Therefore, we also excluded participants who showed above chance-level performance at the lowest contrast level following neutral retrocues. We additionally repeated all our analyses including all volunteers that were tested (see Table A in the S1 File).

### Participants

We tested 37 volunteers (all right-handed according to self-report, mean age 25 years (range 18–35), 12 male). Seven volunteers were excluded from all analyses as they could not perform the task adequately (at chance level in at least one of the conditions). Four additional volunteers were excluded because their performance was outside 2 SD from the group mean. The qualitative pattern of results remained the same whether or not these participants were excluded. An additional six volunteers were excluded because they showed above chance-level performance at 1% contrast following neutral retrocues. The final analyses were performed on data from 20 participants.

### Design

The aim of the first experiment was to examine how the strength of incoming sensory information interacts with top-down orienting of spatial attention within WM to influence performance. Participants completed a total of 480 trials split into 12 blocks of 40 trials each. This yielded 60 trials per condition—valid and neutral retrocue trials at each of four possible Michelson contrast levels: 1%, 18%, 32% and 50%. The lowest contrast level was included with the aim to examine whether attentional selection in WM could raise a stimulus above threshold that would otherwise have been below threshold for WM reports. The remaining three contrast levels were included to examine how contrast level interacted with attentional selection when stimuli are visible (to varying degrees) and reportable.

### Subjective Ratings

On one sixth of trials participants were additionally asked to rate their subjective awareness of the stimuli using the Perceptual Awareness Scale (PAS, [34]. The subjective ratings were included as a secondary measure of awareness (in addition to objective performance) and will not be discussed in this manuscript.

Trials used for subjective report were randomly determined at the beginning of the experiment and randomly spread out across the experiment duration and conditions. Ratings were performed immediately after the feedback and before the interval preceding the successive trial (ITI). The PAS consists of four response options: 1 –stimulus not seen, 2 –weak glimpse: something was there but I do not know its orientation, 3 –almost clear image: I think I know the orientation, and 4 –clear image. Participants were instructed on how to use this scale before the beginning of the experiment. It was emphasized that ratings were to be made introspectively, relying on visual experience [34], and that the ratings were to be made on the initial perceptual, and not the memory representation of the stimuli. A shortened version of the four ratings (1 –stimulus not seen, 2 –weak glimpse, 3 –almost clear image, 4 –clear image) was presented on the screen before ratings were made at the end of the trial. Responses were made by pressing the corresponding number on the keyboard.

### Analysis

For each trial, the reported orientation was collected. This was then measured against the orientation of the probed stimulus to derive recall error and recall precision. Recall error was defined as the angular deviation between the cued orientation and the reported orientation. Recall precision was computed as the trial-to-trial variability in the response error. It was calculated as the reciprocal of the standard deviation (SD) of errors across trials. We used Fisher’s definition of SD for circular data [35], and subtracted the value expected by chance, so that a precision value of zero corresponds to chance-level performance. To avoid confusion, we label the chance-corrected values of SD as SD* in the remainder of the manuscript.

It is important to note that the term recall ‘precision’ can be used to refer to different measurements in WM studies. In some studies (e.g. [24] observers’ WM responses are analysed assuming they reflect a mixture of distributions: a random distribution of responses where observers guess the orientation and a von Mises (circular Gaussian) distribution centred around the target value, reflecting responses related to the target. This distribution is defined by the concentration parameter, or precision, of remembered items (k, or kappa). Some other studies add additional ‘misbinding’ parameters for trials in which observers mistakenly report orientations based on non-target items in the array [30]. The probability of remembering a target is derived from the proportion of random responses. The concentration parameter k is used as an index of representational quality of a WM item, often also referred to as precision. Here, we use the term precision to refer to 1/SD* (as defined above), while we use the term kappa to refer to concentration parameter k as an index of representational quality of items in WM, or WM fidelity. Whereas precision is a measure of overall performance, kappa measures the representational quality when an item is actually encoded, controlling for the frequency of not encoding or remembering an item. This distinction is crucial as our main hypothesis pertains to representational quality in WM.

We examined how retrocueing affects sub-threshold (1% contrast) versus supraliminal (18%–50% contrast) performance using repeated-measures ANOVAs. We averaged over contrast levels 18%–50% to have a measure of performance after sub-threshold versus supraliminal stimulus presentation. Effects of experimental parameters on recall precision were tested using repeated-measures ANOVAs with the factors of retrocue (valid, neutral) and contrast (sub-threshold, supraliminal). Results were followed up by *t* tests to guide interpretation when necessary.

### Results

**Fig 2:**
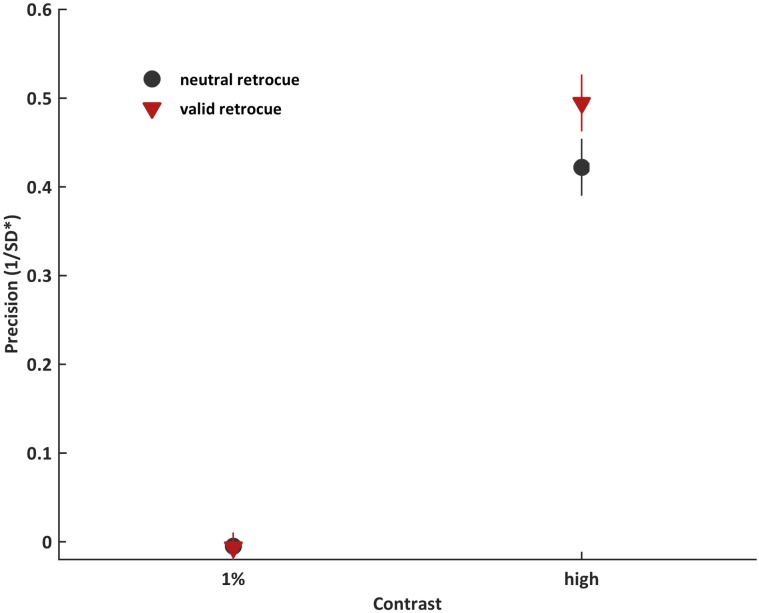
Recall precision for Experiment 1 for sub-threshold (1% contrast) and supraliminal (18%–50% contrast) conditions. Error bars reflect ± 1 standard error of the mean.

Results are presented in Fig 2. The 2-by-2 ANOVA revealed a significant retrocue-by-contrast interaction (F(1,19) = 5.713, *p* = .027, η_p_^2^ = 0.231). There was a retrocueing effect for supraliminal (t(19) = 3.14, *p* = .005, d = 0.702) but not for sub-threshold stimuli (t(19) = 0.06, *p* = .949). Performance was at chance for both 1% conditions (t(19) = 0.355, *p* = .727, *ns*; t(19) = 0.556, *p* = .585, *ns*). Both retrocue (F(1,19) = 6.223, *p* = .022, η_p_^2^ = 0.247) and contrast (F(1,19) = 158.311, *p* < .001, η_p_^2^ = 0.893) also exerted main effects, reflecting that performance was better following valid retrocues and for supraliminal stimulus presentation. These results suggest that retrocueing had a significant effect on recall precision only if stimuli are presented above threshold for WM report.

To examine the effects of contrast on retrocueing more closely, we compared retrocueing effects within supraliminal contrast levels (18%, 32%, and 50%). The 2-by-3 ANOVA revealed a significant main effect of retrocue (F(1, 19) = 9.843, *p* = .005, η_p_^2^ = 0.341), reflecting that performance was better following valid retrocues. However, there was no main effect of contrast (F(2, 38) = 1.423, *p* = .254, *ns*) or retrocue-by-contrast interaction (F(2, 38) = 2.407, *p* = .109, *ns*).

### Interim discussion

The results of Experiment 1 show that top-down attentional orienting during WM maintenance has an effect on recall precision, adding to the growing literature demonstrating the effect of ‘retro-cues’ on WM task performance [1], [2], [12], [36–41]. Our results also reveal that retrocueing effects in WM were modulated by contrast: significant cueing effects only emerged for supraliminal stimuli, but not for sub-threshold stimuli presented at 1% contrast. Once stimuli were presented above threshold, retrocueing effects on recall precision were no longer modulated by contrast level.

In the present study it was not possible to determine which aspects of WM recall were improved following valid cues because we looked at the overall error distribution in the form of recall precision, and did not separate WM responses into different components. Specifically, our results could be explained by changes in the representational quality of a WM item, its likelihood of being remembered, or mistakenly reporting a distracter item instead of the target item. The number of trials per condition prevented us from doing reliable modelling to estimate each of these components. This will be explored in Experiments 2 and 3.

We also found that sub-threshold stimuli did not benefit from attentional orienting: recall performance remained at chance following valid retro-cues. Attention has long been considered to play an integral role in conscious experience [42], [43]. According to Baars’ Global Workspace Theory [42] neural activation elicited by a stimulus needs to be amplified by top-down attentional signals for a stimulus to be consciously experienced. According to this account, attention has a direct role in shaping and strengthening unconscious representations. Our findings using retrocues could reflect one of two things. One possibility is that mechanisms are different for WM. For example, weak, sub-threshold representations may not remain long enough to be boosted by attention. Varying the time point at which the retrocue is presented during the WM delay period could test this hypothesis (see also [27], [33]. Alternatively, the neural activity elicited by the 1% contrast stimulus was too weak to exceed a threshold even after attentional boosting, for example if the stimulus was not perceived or encoded at all.

## Experiment 2

The aim of this follow-up experiment was to separate the effects of cueing and contrast on the different distributions making up the overall WM error distribution. To this end, we doubled the number of trials so we could perform mixture modelling to estimate the different sources of error [30]. Participants completed a total of 480 trials split into 12 blocks of 40 trials each (120 trials per condition). Only two contrast levels were used for the stimulus array– 18% and 50%–resulting in more trials per condition for modelling of the behavioural results. We did not collect subjective ratings.

### Inclusion Criteria

We only included participants who performed within 2 SD of the group mean in all conditions (not averaged over contrast).

### Participants

We tested 32 volunteers (all right-handed according to self-report, mean age 26 years (range 18–35), 10 male). Two volunteers were excluded from all analyses as they could not perform the task adequately (at chance level in at least one of the conditions). After also excluding volunteers with performance outside 2 SD from the group mean, the following numbers of participants were entered into the final analyses: recall precision N = 27; target recall probability N = 28; misbinding N = 25; guess rate N = 27, and kappa N = 24. The qualitative pattern of results remained the same if these participants were included.

### Analysis

For each trial, the reported orientation was collected. This was then measured against the orientation of the probed stimulus to derive recall error and recall precision. We modelled different sources of errors contributing to memory performance by applying a probabilistic model to the data [30]. The model attributes the overall distribution of responses to a mixture of three possible sources of errors on each trial: 1) a von Mises distribution (circular analogue of the Gaussian) centred on the target direction, 2) a uniform distribution of error corresponding to random responses, and 3) von Mises distributions centred around each of the non-target orientations in a given stimulus display, i.e. a proportion of errors that corresponds to participants mistakenly reporting an orientation based on a non-target item. This model is described by the following equation:

$$p(\hat{\theta })=\alpha \Phi_{\kappa }(\hat{\theta }-\theta )+\;\beta \;\frac{1}{m}\;\Sigma_{i}^{m}\Phi_{\kappa }(\hat{\theta }-\varphi_{i})+\;\gamma \;\frac{1}{2\pi }$$

where θ is the actual orientation of the target, $\hat{\theta }$ is the reported orientation,*Φ*_*κ*_ is the von Mises distribution with mean zero and concentration parameter *κ*. The concentration parameter *κ* corresponds to the variability of target recall, where greater *κ* corresponds to lower variability in the distribution. We refer to it as kappa and use it as an index of representational quality of WM items. The probability of reporting the targets, or the probability of remembering the target item, is given by *α*. The probability of reporting a distracter is given by *β* which we use as an index of misbinding, and *φ*_1_, *φ*_2_, …, *φ*_*m*_ are the orientations of the *m* non-target items. The probability of randomly responding (or guessing) is given by γ = 1 − α − *β*.

Maximum-likelihood estimates of each parameter (α, β, γ, and κ) were obtained by using an expectation-maximization algorithm separately for each participant and experimental condition [30].

We examined the effects of experimental parameters on recall precision, kappa, misbinding, the probability of target recall, and guess rate at the group level using repeated-measures ANOVAs with the factors of retrocue (valid, neutral) and contrast (18%, 50%). Additional *t* tests (Bonferroni corrected where appropriate) were run when necessary.

### Results

**Fig 3:**
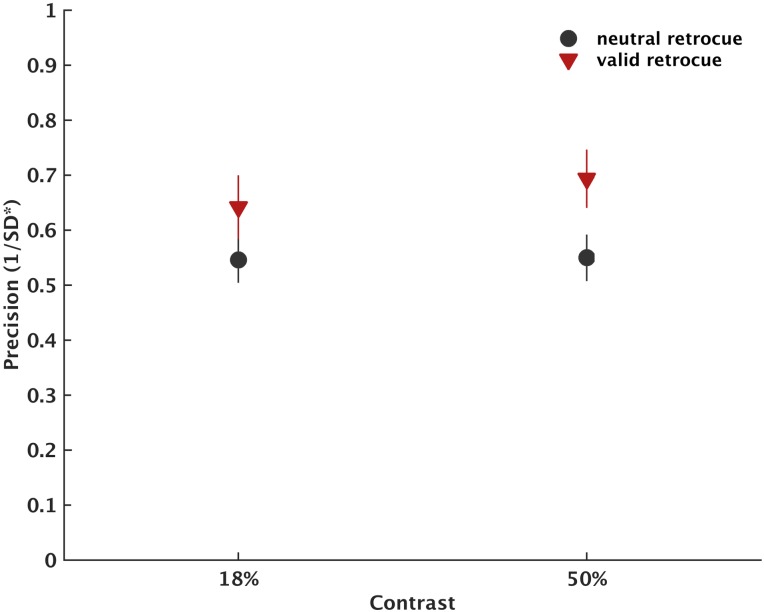
Recall precision for each condition separately of Experiment 2. Error bars reflect ± 1 standard error of the mean.

**Fig 4:**
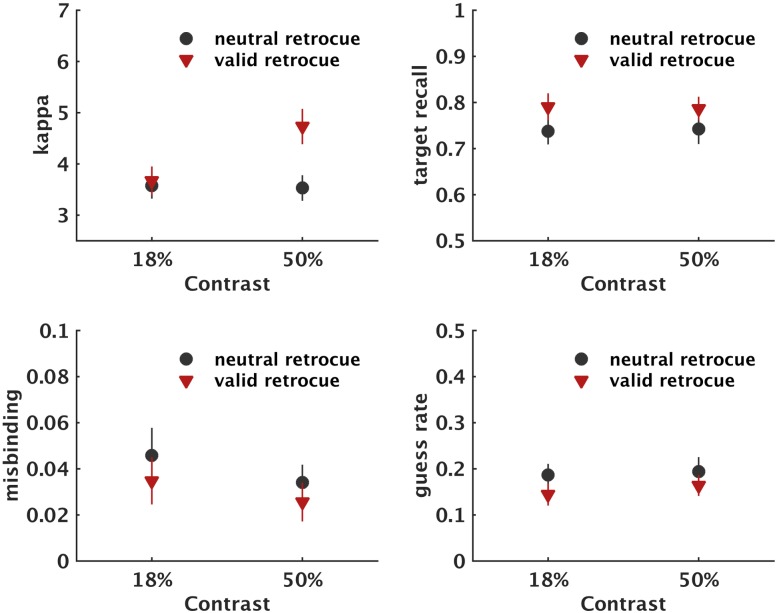
Modelling parameters for each condition separately of Experiment 2. Error bars reflect ± 1 standard error of the mean.

Results are presented in Figs 3 and 4. Retrocue validity exerted a significant main effect on recall precision (F(1,26) = 23.04, *p* < .001, η_p_^2^ = 0.470). There was no significant effect of contrast (F(1,26) = 2.72, *p* = .111, *ns*), and the two factors did not interact (F(1,26) = 1.449, *p* = .239, *ns*). These results replicated the effect of retrocue validity reported in Experiment 1, where recall precision was better following valid compared to neutral cues, and this did not change with stimulus contrast as long as stimuli were presented above threshold.

For kappa, there was a significant interaction between retrocue and contrast (F(1,23) = 14.647, *p* = .001, η_p_^2^ = 0.389). The main effects of retrocue and contrast were also significant (F(1,23) = 7.721, *p* = .011, η_p_^2^ = 0.251 and F(1,23) = 14.111, *p* = .001, η_p_^2^ = 0.380, respectively). Kappa was higher following valid compared to neutral cues but only at 50% contrast (*t*(23) = 3.99, *p* < .001, d = 0.82) and not at 18% contrast (*t*(23) = 0.37 *p* = .718, d = 0.07).

For target recall probability there was a significant main effect of retrocue (F(1,27) = 8.87, *p* = .006, η_p_^2^ = 0.247). Observers reported the target orientation significantly more often following valid compared to neutral cues. The main effect of contrast was not significant (F(1,27) = 0.004, *p* = .952, *ns*) and the two factors did not interact (F(1,27) = 0.138, *p* = .713, *ns*).

For misbinding, the main effect of retrocue (F(1,24) = 1.466, p = 0.238, *ns*), the main effect of contrast (F(1,24) = 2.026, p = 0.168, *ns*), and the retrocue-by-contrast interaction (F(1,24) = 0.072, p = 0.791, *ns*) were not significant. Similarly, for guess rate the main effect of retrocue (F(1,26) = 3.774, p = 0.063, *ns*), the main effect of contrast (F(1,26) = 0.772, p = 0.388, *ns*), and the retrocue-by-contrast interaction (F(1,26) = 0.149, p = 0.703, *ns*) were not significant.

### Interim discussion

As in Experiment 1, the results showed that recall precision was improved following valid retrocues. The cueing benefit on WM recall precision was not modulated by contrast levels. This supports findings from Experiment 1 in which we also found that baseline precision is not strongly modulated by contrast once Gabor patches were presented at a contrast level that enabled observers to perform above chance-level.

The ability to model the data with the increased number of trials allowed us to understand what variables contributed to the changes in performance. Specifically, we showed that the overall improvement in recall precision following valid retrocues involved an increase in the representational quality of WM items (kappa) at high contrast levels and an overall increase in the probability of remembering the target item independently of contrast. The proportion of misbinding errors and guessing rates were not modulated by retrocue validity or contrast.

In Experiments 1 and 2 we did not observe an influence of supraliminal stimulus contrast on baseline WM performance (i.e. in neutral retrocueing trials). The contrast levels used in both experiments may have been beyond any effects of facilitation, and performance never approached the lower asymptote of the psychometric curve. Instead, performance was at chance at 1% contrast and had already reached a plateau at 18% contrast. One possible interpretation is that memory for perceived orientations is categorical, and a stimulus is remembered at a representational quality independent of its quality during sensory encoding as long as it is presented above threshold. Alternatively, it is still possible that memory quality is affected by the sensory quality of the stimulus during encoding, but that the contrast levels used in the previous two studies might have missed the range of any facilitation effect. Similarly, relative patterns of misbinding and kappa, and cueing effects on these parameters, may change as performance approaches the lower asymptote.

Experiments 1 and 2 gave us no leverage on the question of whether graded effects of contrast might be observed with low contrast levels. To explore whether retrocues rely on different mechanism as stimulus contrast transitions from sub-threshold to at-threshold and on to clearly visible, and to investigate how such changes might affect memory recall following neutral retrocues, we examined performance with low contrast levels (between 1% and 18%) in greater detail in Experiment 3. An alternative to using set contrast levels would have been to titrate contrast levels based on individual psychophysical thresholds. We chose not to do this because it is not straightforward to translate results from a standard forced-choice staircasing procedure to the WM task used here.

## Experiment 3

Building on the findings of the first two experiments, the third experiment sampled the contrast-level space in between the sub-threshold and low contrast levels more extensively (3%, 10%, and 18%). A second aim of the Experiment was to characterise patterns of kappa and misbinding at lower levels of contrast. As in Experiment 2, large trial numbers were used to enable modelling of the data to reveal the pattern of effects across kappa, target response probability, misbinding and proportion of guessing. Participants completed a total of 600 trials split into 15 blocks of 40 trials each (100 trials per condition). In addition, eye gaze was tracked to ensure that differences between conditions were not related to differences in eye movements.

### Inclusion Criteria

We only included participants who performed within 2 SD of the group mean in all conditions (not averaged over contrast).

### Participants

We tested 38 volunteers (all right-handed according to self-report, mean age 24 years (range 18–35), 12 male). Four volunteers were excluded from all analyses because they could not perform the task adequately (at chance level in at least one of the conditions After also excluding volunteers with performance outside 2 SD from the group mean, the following numbers of participants were entered into the analysis: recall precision N = 31; target recall probability N = 30; misbinding N = 28; guess rate = 29 and kappa N = 28. The qualitative pattern of results remained the same if these participants were included.

### Analysis

The analysis approach followed that of Experiment 2. We examined effects of experimental parameters on recall precision, kappa, misbinding, the probability of target recall, and guess rate using repeated-measures ANOVAs with factors of retrocue (valid, neutral) and contrast (3%, 10%, 18%). Follow-up *t* tests clarified patterns of effects where needed (Bonferroni corrected where appropriate). When eye-tracking data were available, trials in which eyes moved more than 2 degrees from fixation were removed from all analyses (~6% trials). For seven participants eye-tracking data were not available due to technical difficulties during recording.

### Results

**Fig 5:**
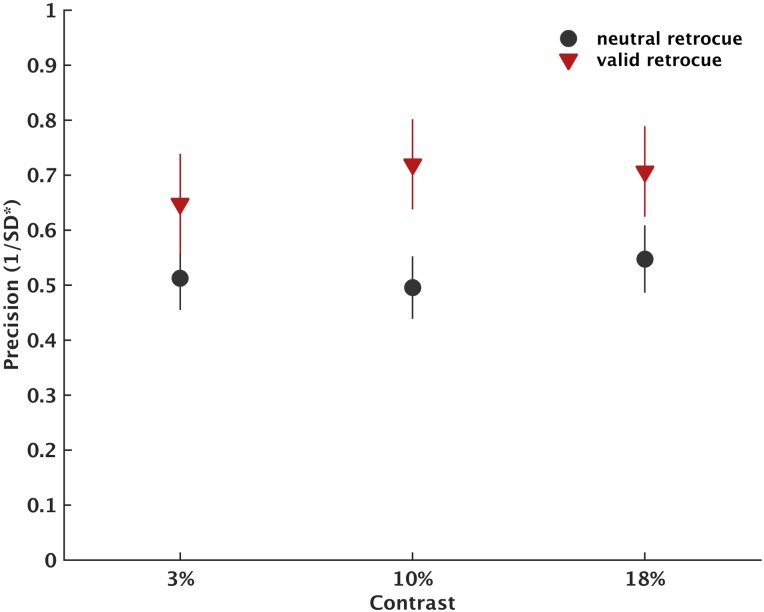
Recall precision for each condition separately of Experiment 3. Error bars reflect ± 1 standard error of the mean.

**Fig 6:**
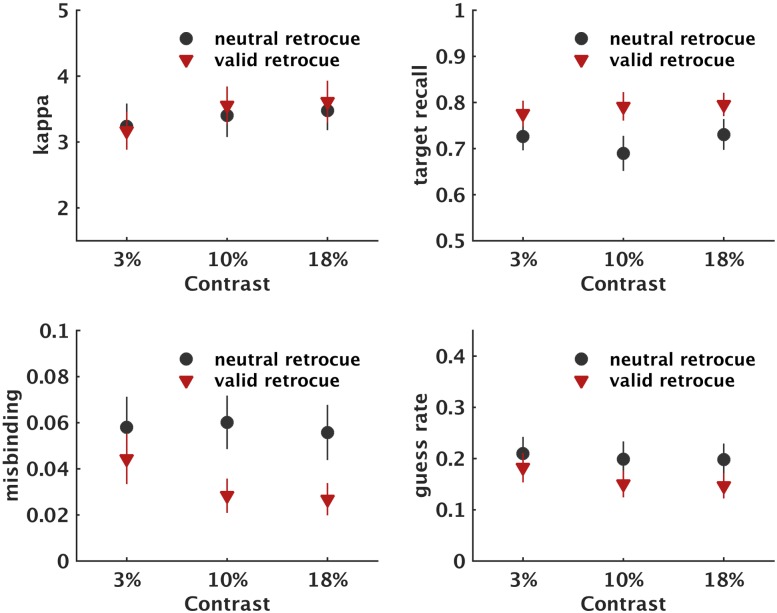
Modelling parameters for each condition separately of Experiment 3. Error bars reflect ± 1 standard error of the mean.

Results are presented in Figs 5 and 6. For recall precision, there was a significant retrocue-by-contrast interaction (F(2,60) = 3.878, *p* = .0028, η_p_^2^ = 0.114). To examine the interaction more closely, we ran follow-up *t* tests comparing recall precision following valid-retrocue and neutral-retrocue trials at each of the three contrast levels. These revealed significant retrocueing effects at contrast levels 3% (t(30) = 3.043, *p* = .005, d = 0.547), at 10% (t(30) = 4.614, p < .0001, d = 0.829) and at 18% (t(30) = 3.944, p < .001, d = 0.708). However, cueing effects were significantly smaller at 3% contrast compared to 10% contrast (t(30) = 2.780, *p* = .009, d = 0.499) but not different to cueing effects at 18% (t(30) = 1.906, p = .066, *ns*). Cueing effects at 10% were not different to cueing effects at 18% (t(30) = 0.926, *p* = .362, ns; CueingEffect_3%_ = 0.0633 ± 0.01; CueingEffect_10%_ = 0.16 ± 0.02; CueingEffect_18%_ = 0.126 ± 0.02). In other words, cueing effects increased from 3% to 10% and then remained stable. We also observed significant main effects of retrocues (F(1,30) = 29.447, *p* < .001, η_p_^2^ = 0.495) and contrast (F(2,60) = 6.506, *p* = .005, η_p_^2^ = 0.178).

For kappa, the main effect of retrocue, the main effect of contrast, and the retrocue-by-contrast interaction were not significant (F(2,54) = 1.144, p = 0.320, *ns*, and F(2,54) = 0.184, p = 0.789, *ns*; respectively).

For target recall probability there was a significant main effect of retrocue (F(1,29) = 13.994, *p* = .001, η_p_^2^ = 0.326). Observers reported the target orientation significantly more often following valid compared to neutral retrocues. The main effect of contrast and the contrast-by-retrocue interaction were not significant (F(2,58) = 0.683, p = .495, *ns*, and (F(2,58) = 1.138, p = .326, *ns*; respectively).

For misbinding there was a significant main effect of retrocue (F(1,27) = 10.386, *p* = .003, η_p_^2^ = 0.278). Observers reported the non-target orientation significantly more often following neutral compared to valid retrocues. The main effect of contrast (F(2,54) = 0.520, p = .570, *ns*) and the retrocue-by-contrast interaction (F(2,54) = 0.607, p .545, *ns*) were not significant.

For guess rate, the main effect of contrast (F(1,28) = 4.069, p = .053, *ns*), the main effect of contrast (F(2,56) = 4.069, p = .501, *ns*) and the retrocue-by-contrast interaction (F(2,54) = 0.191, p = .814, *ns*) were not significant.

### Interim discussion

As in Experiments 1 and 2, the results showed that recall precision is better following valid compared to neutral retrocues. A novel finding is that for the contrast levels used in this study (3, 10 and 18%) retrocueing effects were modulated by contrast level. Specifically, retrocueing effects on recall precision increased from 3% contrast to 10% contrast and then remained stable.

Applying a mixture model to the data allowed us to examine the pattern of results more closely. The effects on target response probability and guess rate were comparable to Experiment 2: target-response probability was increased while guess rate was unaffected following valid retrocues. The kappa results are also in line with the low-contrast findings from Experiment 2 and we observed no effect of retrocueing at or below 18% contrast. Together, this suggests that retrocues only affect kappa, i.e. the representational quality of WM items, at high levels of contrast.

The use of low contrast stimuli in Experiment 3 revealed an effect of retrocueing on misbinding rates, with decreasing misbinding levels in valid compared to neutral retrocueing trials. Overall higher levels of misbinding in Experiment 3 compared to Experiment 2 under neutral retrocueing conditions (although not significantly, M_E2_ = 4% ±0.8%, M_E3_ = 5.8% ±0.7%, p = 0.103) may have provided a margin to reveal performance benefits in this function.

## General Discussion

Over the three experiments, our results indicate that the quality of incoming sensory information interacts with attentional orienting to shape the representational quality of items in WM. Retrocueing effects on overall recall precision were modulated by the quality of incoming sensory information and were more pronounced at higher levels of contrast, specifically at contrast levels above 10% compared to contrasts at or below 3%. Using a model-based approach we found evidence that the process by which retrocueing affects WM recall is influenced by the quality of incoming sensory information. While effects of retrocueing on target recall and guess rate were relatively constant across Experiment 2 and 3, and across different contrast levels within experiments, effects of retrocueing on kappa and misbinding differed between experiments and contrast levels. At high levels of contrast (Experiment 2) effects of retrocueing were observed on kappa, while at low levels of contrast (Experiment 3) retrocueing effects were revealed on misbinding rates.

The relative proportion of misbinding often varies between studies ranging from no misbinding up to 30% [17]. [17] recently proposed a formal account of misbinding errors. According to this model, representations for different WM items overlap because one population of neurons encodes all items. WM performance is limited by neural noise and item overlap, and how items overlap is determined by how items are encoded. Overlapping items can lead to misbinding errors as items interfere with one another. The balance between two kinds of units, which either store individual features or binding information, determines the relative proportion of misbinding errors and the representational quality of items in WM (kappa). However, this model does not address how changes in noise modulate misbinding versus kappa. A model proposed by [16] attributes WM recall errors to the presence of noise in the neural populations that code WM items. Noise limits the decodability of neural populations and item-related activity diffuses over time. Increases in noise can be countered by increases in gain, which amplifies the strength of the coding signal. Although this model only considers internal but not external sources of noise, it does provide a framework to consider the effects of relative signal to noise levels during encoding on representational quality of WM items.

Combining these two models allows us to offer tentative explanations on how noise fluctuations and attentional mechanisms interact to shape the representational quality of, and relative misbinding rates between, items in WM. Our results support the suggestion that overlap and interference between items in WM depends on how they are encoded [17]. When the quality of sensory input is low, such as when items are presented at low contrast, items are encoded with more overlap and interference between items increases. Attentional selection (e.g., from retrocueing) then increases the gain and signal-to-noise ratio (SNR) of individual representations, separating noisy, overlapping items. This allows for categorical distinctions to be drawn between representations, reducing the potential for misbinding errors. At higher contrast levels with a higher SNR, different items are encoded with less overlap and attentional selection does not affect item-overlap as items are already (relatively) readily distinguished—at least in the experimental paradigm employed here. Attentional selection and increases in gain do not further differentiate item representations but instead the increase in gain sharpens item representations, thereby increasing the quality of memory recall (as measured by the concentration parameter, kappa).

The proposal that retrocueing mechanisms are flexible and depend on the representational quality of WM items, as determined, for example, by stimulus properties during encoding, also helps to resolve an apparent contradiction between our high-contrast results with claims from a previous study that the retrocueing benefit is due to an increased probability of correctly reporting the target item, rather than an increase in fidelity [4]. While [4] used high contrast stimuli, the set size (4 or 8 items) was much larger than in our experiment and orientation stimuli covered 360 degrees (as opposed to 180 degrees in our study). Despite the use of high contrast stimuli the representational quality of WM items in [4] is therefore unlikely to be comparable to our study, as task demands were different. Overall, these findings are in line with our argument that retrocueing mechanisms are flexible and depend on task specifics (see [5] for a similar argument).

It may seem counterintuitive that attention could increase SNR retrospectively, selectively increasing signal without any additional information regarding what is signal and what is noise. There are a number of scenarios under which precision of memoranda may be selectively enhanced. One possible implementation might be based on the ‘Bell and Hammer’ model [44]. According to this model, WM traces in sensory cortices are periodically reactivated before the activity returns to baseline noise levels. Neurons participating in memory representations can be selectively targeted by attention because they have higher levels of activity than nearby neurons (assuming the memory trace has not decayed yet). In line with this model, the brain ‘knows’ what is signal and what is noise on the basis of relative activity levels, even without receiving new input. Another possibility is that retrocues improve WM representations by using information not encoded in neural activity, but in ‘activity-silent’ formats such as changes in the weights of functional connections [45], as was recently proposed by [46]. The authors used image reconstruction techniques on fMRI data to assess the fidelity of WM representations in different conditions and found that valid retrocues can recover the fidelity of degraded WM representations. The amplitude of WM representations was higher in 2-item valid retrocue trials compared to 2-item neutral retrocue and 1-item trials. These findings suggest that WM representations are not permanently impaired, and information is not permanently lost, but instead can be recovered using for example retrocues, in line with our conclusions. If WM representations were based solely on active codes such as spiking activity, mutual suppression between WM representations would lead to permanent information loss. In contrast, ‘activity-silent’ sources of information can be used to improve representations without such suppressive interactions. There may yet be additional possibilities. For example, object-related representations may involve distributed coding in neuronal ensembles that still compete with one another in WM, and attention (much in the same way proposed for perceptual analyses) may prioritise information about one item. This could lead to suppression of information from other items and enhancement of information related to the prioritized items in order for access to the qualities of that representation to be better integrated and suffer less interference than would otherwise be possible. Future studies are needed to characterize further the relative influences of the quality of incoming sensory information and attentional selection on kappa and misbinding rates, and the underlying mechanisms.

## Supporting Information

S1 File Additional analyses results for Experiments 1–3. P-values for Experiments 1–3 when all volunteers were included in the analysis. Importantly, the results remained (largely) equivalent. The critical cueing effect on kappa in Experiment 2 but not Experiment 3, and on misbinding in Experiment 3 but not 2, remained significant regardless of exclusion criteria.
(DOCX)
