# Supplementary material for: Retrospective Attention Interacts with Stimulus Strength to Shape Working Memory Performance
Source: PLoS One. 2016 Oct 5;11(10):e0164174. doi: 10.1371/journal.pone.0164174 (PMC5051714; doi:10.1371/journal.pone.0164174)
Supplement: S1 File — P-values for Experiments 1–3 when all volunteers were included in the analysis. Importantly, the results remained (largely) equivalent. The critical cueing effect on kappa in Experiment 2 but not Experiment 3, and on misbinding in Experiment 3 but not 2, remained significant regardless of exclusion criteria. (DOCX) [file pone.0164174.s001.docx]

**Supporting Information**

Table A: P-values for Experiments 1-3 when all volunteers were included in the analysis. Importantly, the results remained (largely) equivalent. The critical cueing effect on kappa in Experiment 2 but not Experiment 3, and on misbinding in Experiment 3 but not 2, remained significant regardless of exclusion criteria.

|  | Main effect cue | Main effect contrast | Cue * contrast |
| --- | --- | --- | --- |
| Experiment 1 |  |  |  |
| Recall precision | F( 1.00,36.00) = 13.679, p = 0.001 | F( 1.00,36.00) = 92.426, p = 0.000 | F( 1.00,36.00) = 13.932, p = 0.001 |
| Experiment 2 |  |  |  |
| Recall precision | F( 1.00,31.00) = 19.873, p = 0.000 | F( 1.00,31.00) = 3.233,p = 0.082 | F( 1.00,31.00) = 3.294, p = 0.079 |
| Kappa | F( 1.00,31.00) = 9.260, p = 0.005 | F( 1.00,31.00) = 11.546, p = 0.002 | F( 1.00,31.00) = 4.284, p = 0.047 |
| Target recall | F( 1.00,31.00) = 2.268, p = 0.142 | F( 1.00,31.00) = 0.037,p = 0.848 | F( 1.00,31.00) = 1.147, p = 0.292 |
| Misbinding | F( 1.00,31.00) = 0.550, p = 0.464 | F( 1.00,31.00) = 1.440,p = 0.239 | F( 1.00,31.00) = 0.466, p = 0.500 |
| Guess rate | F( 1.00,31.00) = 1.082, p = 0.306 | F( 1.00,31.00) = 0.473,p = 0.497 | F( 1.00,31.00) = 1.046, p = 0.314 |
| Experiment 3 |  |  |  |
| Recall precision | F( 1.00,37.00) = 28.090, p = 0.000 | F( 2.00,74.00) = 3.090,p = 0.062 | F( 2.00,74.00) = 1.774, p = 0.179 |
| Kappa | F( 1.00,37.00) = 0.000, p = 1.000 | F( 2.00,74.00) = 1.752,p = 0.186 | F( 2.00,74.00) = 0.060, p = 0.905 |
| Target recall | F( 1.00,37.00) = 12.893, p = 0.001 | F( 2.00,74.00) = 0.986,p = 0.370 | F( 2.00,74.00) = 1.134, p = 0.324 |
| Misbinding | F( 1.00,37.00) = 4.305, p = 0.045 | F( 2.00,74.00) = 0.030,p = 0.958 | F( 2.00,74.00) = 0.514, p = 0.591 |
| Guess rate | F( 1.00,37.00) = 4.810, p = 0.035 | F( 2.00,74.00) = 0.505,p = 0.578 | F( 2.00,74.00) = 0.424, p = 0.646 |
